# The British Journals: Materia Medica and Chemistry

**Published:** 1839-10

**Authors:** 


### MATERIA MEDICA, CHEMISTRY.

*Economic Formula for Hydriodate of Potash.* By WM. NICHOLS, M.R.C.S.

RUB together as much iodine and potass hydras (the potassa fusa of the former Pharmacopœia) as will render the mixture almost colourless, and add as much distilled water as will make, together, say, two fluid ounces.

The chemical equivalents of the iodine and potassa would of course be the proper proportions, provided they could be obtained perfectly pure, which, in commerce, I believe to be seldom the case. I therefore choose to get my solution prepared as above, of an amber colour, showing the iodine to be slightly in excess, and I afterwards add a few drops of the liq. potassæ, until the solution becomes perfectly colourless. By previously weighing the proportions of solid ingredients, the quantity of the salt in solution will be indicated; and as it is extremely soluble, it may be prepared so that each fluid drachm will contain a drachm of the hydriodate. *Lancet. Aug.* 25, 1839.

